# Supplementary material for: Cryo-EM structure of the polycystic kidney disease-like channel PKD2L1
Source: Nat Commun. 2018 Mar 22;9:1192. doi: 10.1038/s41467-018-03606-0 (PMC5864754; doi:10.1038/s41467-018-03606-0)
Supplement: Supplementary file 1 — Supplementary Information(PDF 4169 kb) [file 41467_2018_3606_MOESM1_ESM.pdf]

Cryo-EM structure of the polycystic kidney disease-like channel PKD2L1

Su et al.

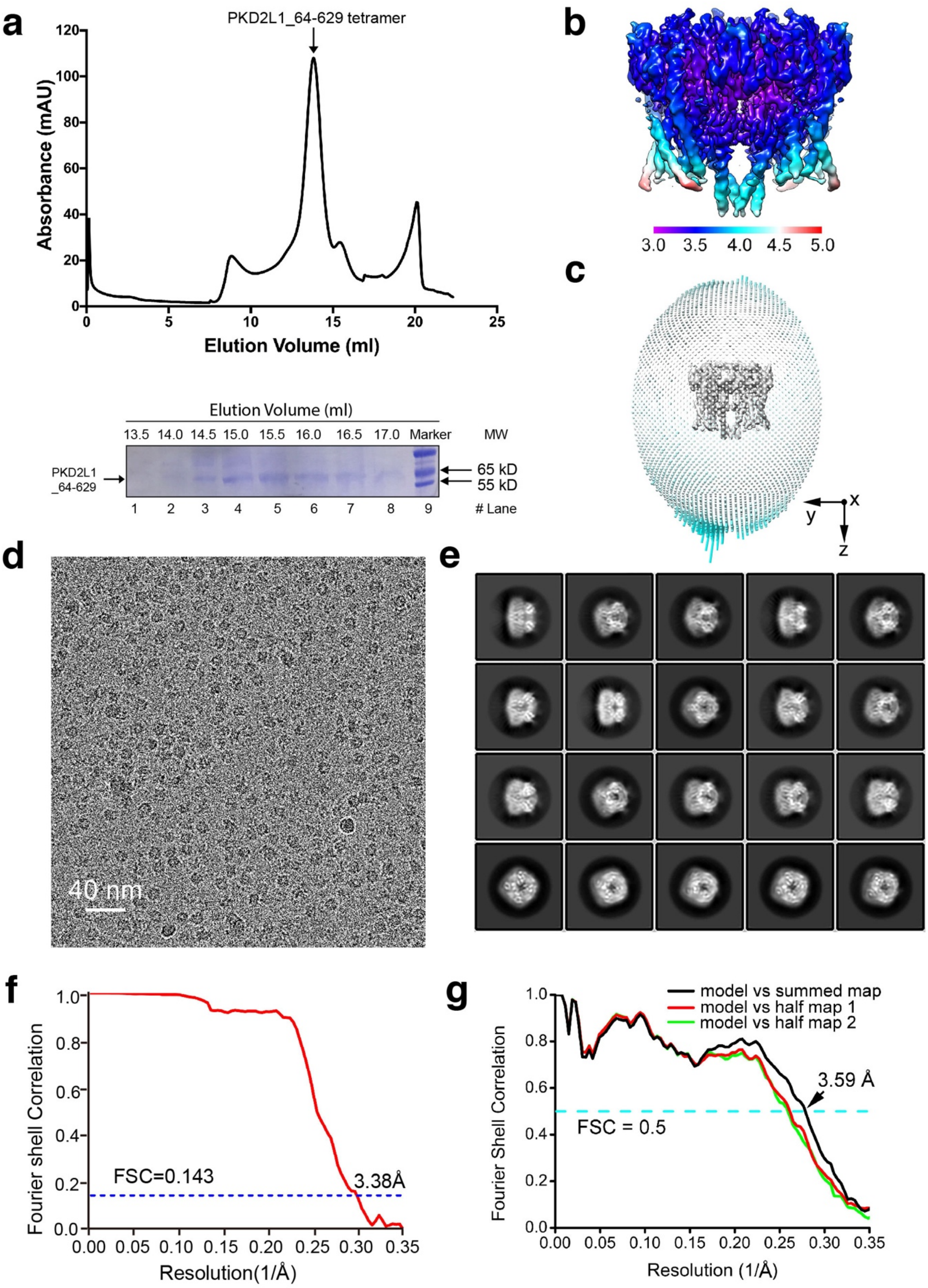

Supplementary Figure 1 Cryo-EM analysis of PKD2L1 (residues 64–629). (**a**) Size exclusion chromatogram and SDS-polyacrylamide gel electrophoresis of PKD2L1 (residues 64–629) in amphipols.

The peaks corresponding to the channel are indicated with arrows. (**b**) Local resolution estimates determined by ResMap; shown from the side view. (**c**) Angular distribution of particles for final reconstructions. Each cylinder represents one view and the size of the cylinder is proportional to the number of particles for that view. The azimuth angle only spans 90° because of the fourfold symmetry axis. (**d**) Representative electron micrographs of PKD2L1 (residues 64–629). (**e**) 2D class averages of selected particles of PKD2L1 (residues 64–629). (**f**) Gold-standard FSC curves for EM maps, after RELION auto-refinement. (**g**) FSC curves for cross-validation between the model and the cryo-EM maps. Shown here are the FSC curves between the final refined atomic model and the reconstruction from all particles (black), between the model refined in the reconstruction from only half of the particles and the reconstruction from that same half (red), and between that same model and the reconstruction from the other half of the particles (green).

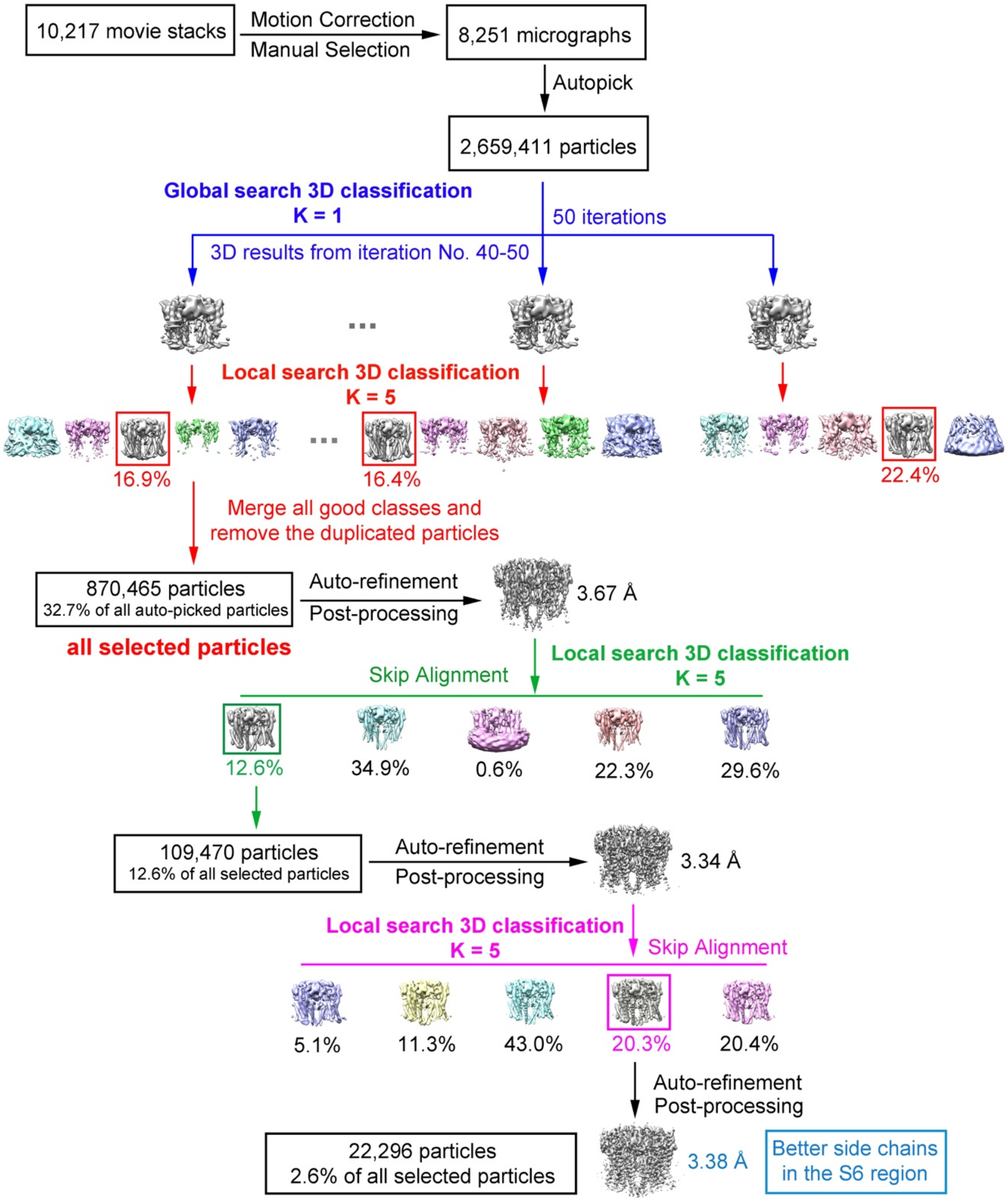

Supplementary Figure 2 Flowchart for EM data processing of PKD2L1 (residues 64–629). Details can be found in the Methods section.

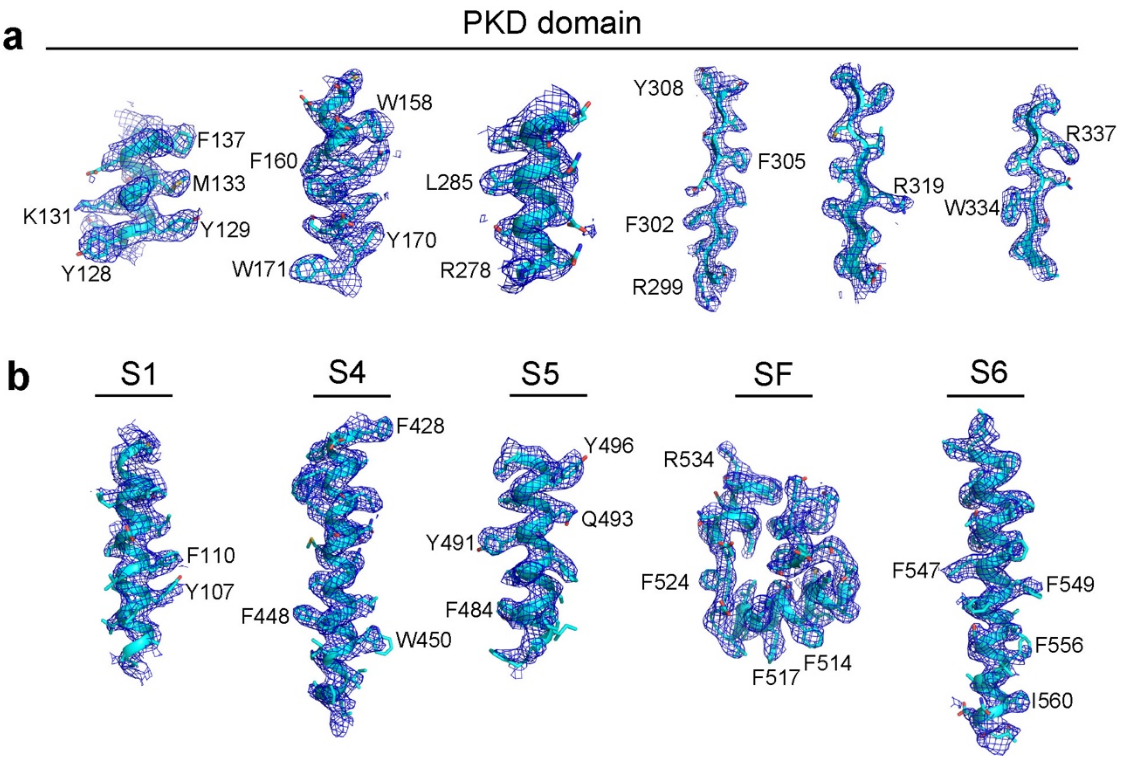

Supplementary Figure 3 Representative local EM maps of PKD2L1 (residues 64–629). (**a**) EM maps for representative segments in the PKD domain in PKD2L1 (residues 64–629). (**b**) EM maps for representative segments in the transmembrane domain of PKD2L1 (residues 64–629). The atomic models of PKD2L1 (residues 64–629) are colored in cyan. The maps, shown as blue mesh, are contoured at 4–5 σ and generated in PyMOL (The PyMOL Molecular Graphics System, Version 1.8 Schrödinger, LLC). The representative bulky side chains that were used to aid sequence assignment are labeled. SF represents the selectivity filter area.

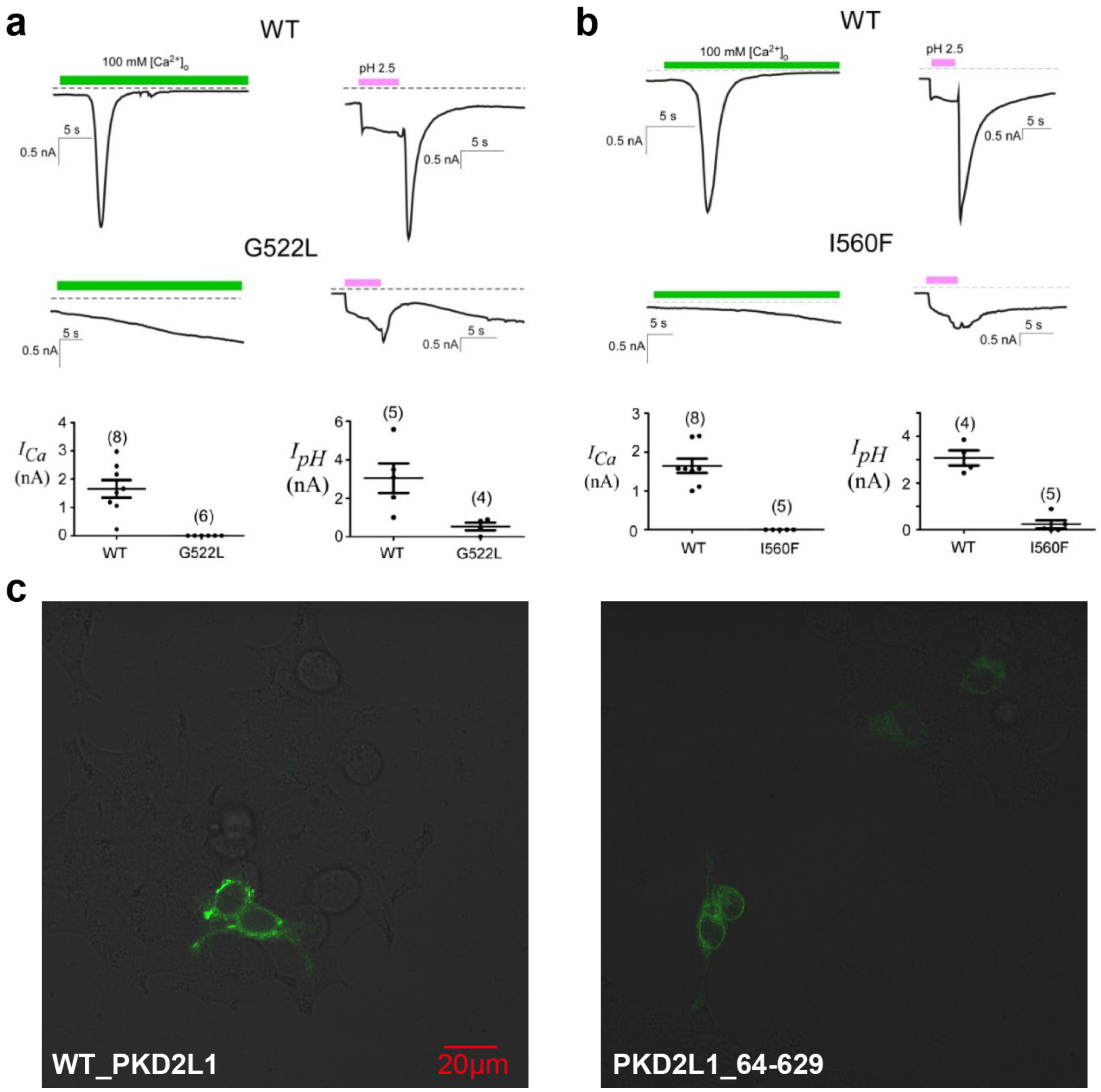

Supplementary Figure 4 Mutations of two major constriction sites along the pore and confocal images. (**a**) Mutations at the constriction site of the outer pore. Compared with WT channels, mutant G522L channels failed to produce any observable currents of I_{Ca} or I_{pH}. (**b**) Mutations at the constriction site of the lower gate. Large I_{Ca} and I_{pH} were induced from WT PKD2L1, but not the mutant I560F. All above values in (a) and (b) are in mean±SEM, indicated with significance (*, p<0.05; **, p<0.01; and ***, p<0.001). (**c**) Merged confocal pictures (of bright field and YFP) showing the membrane trafficking of both wild-type PKD2L1 (WT_PKD2L1) and truncated PKD2L1 (PKD2L1_64-629), both in complex with PKD1L3. Experiments were conducted in the same day and were repeated for several times with average performance.

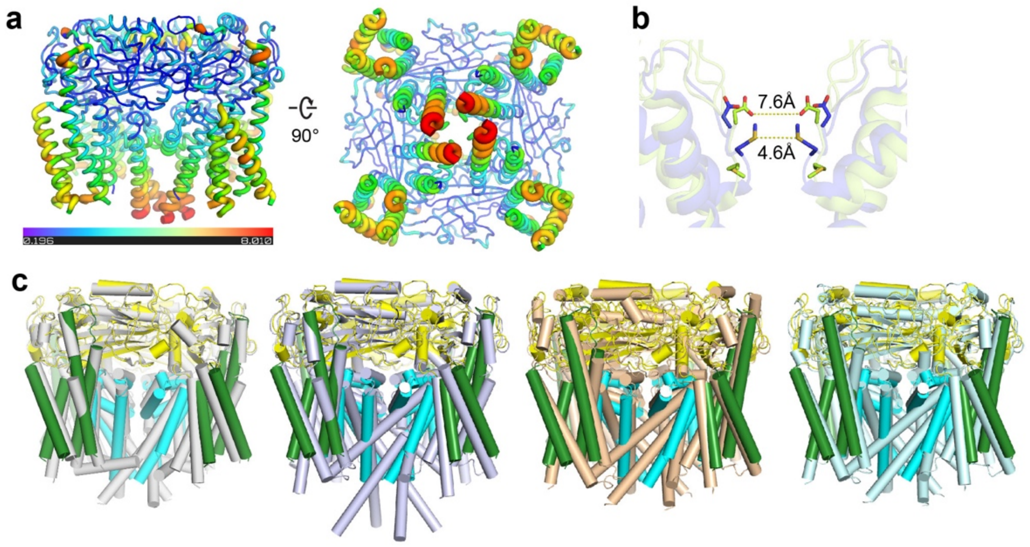

Supplementary Figure 5 Topological comparisons of PKD2L1 and PKD2[1,8,9]. (**a**) Structural differences between PKD2L1 and PKD2 following RMSD analysis. The structure of PKD2L1 (residues 64–629) is shown in the form of tubes from two views. RMSD is calculated with respect to Cα and indicated by the color and radius of the tubes. As shown in the model, polycystin domains exhibit minimal differences. (**b**) Dilation of the selectivity filter in TRPV1 from 4.6 Å (PDB code: 5IRZ, lime) to 7.6 Å (PDB code: 5IRX, blue) when transitioning from an apo-closed state to a fully opened conformation[10]. (**c**) Structural comparison of PKD2L1 to four published structures of PKD2: 5T4D (gray), 5K47 (bluewhite), 5MKE (wheat) and 5MKF (pale cyan) respectively. All structures are superimposed holistically and shown as cylindrical models.

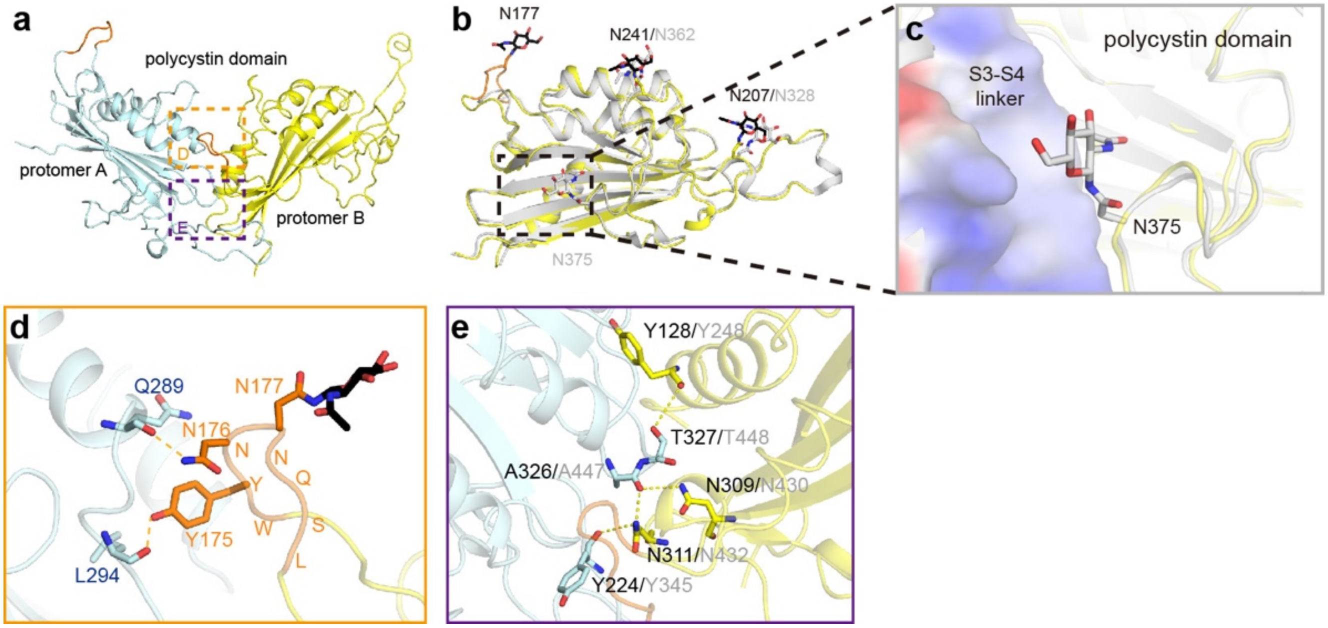

Supplementary Figure 6 Structural analysis of polycystin domains between PKD2L1 and PKD2. (**a**) Interface between two neighboring polycystin domains of PKD2L1. A unique visible loop in PKD2L1 of seven residues is shown in orange. (**b**) Different glycosyl sites in PKD2L1 (yellow, black) and PKD2 (gray, gray) are shown as sticks. Each protomer of PKD2L1 has three glycosylation sites: N177, N207, and N241 whereas the three glycosylation sites of PKD2 are N328, N375, and N362. (**c**) In the PKD2 structure, N375 helps stabilize the inter-domain interactions with respect to the S3-S4 linker. Respectively, the absence of this specific asparagine and the corresponding oligosaccharide chain in PKD2L1 may impact the interactions between PKD2L1 S3-S4 linkers and its polycystin domains. (**d**) A portion of density is invisible in the PKD2 structure, corresponding to the seven residues between 296 and 302, whereas it is clearly evidenced in the PKD2L1 structure (aa. 174–180). These seven residues appear as a loop. This loop interacts with the adjacent polycystin domain by two pairs of hydrogen bonds (Q289 and N176, Y175 and L294), enhancing the assembly of polycystin domains and the overall stability. (**e**) Zoomed view indicating the similar interactions of PKD2L1 or PKD2 (PDB code: 5T4D) at the interface.

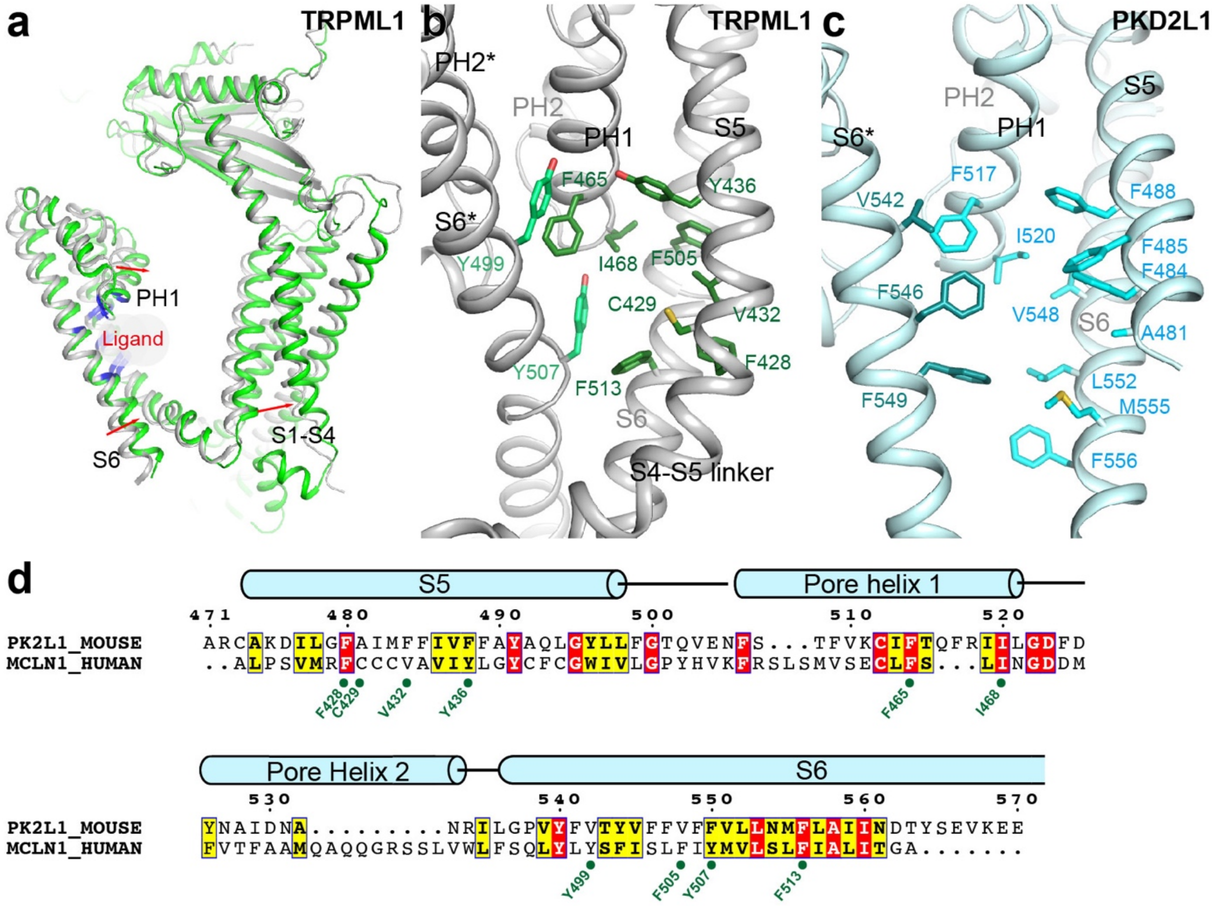

Supplementary Figure 7 Structural analysis of TRPML1's ligand-induced conformational changes (closed-state TRPML1 shown as gray, PDB code: 5WJ5; open-state TRPML1 shown as bright-green, PDB code: 5WJ9)[11]. (**a**) Structural comparisons of one protomer between TRPML1's two states indicate its potential regulatory mechanisms. The red arrows represent overall shifts from the closed to open state upon ligand binding. A slight outward movement of PH1 associates distinct dilations of the lower gate, together with some minor structural changes of S1-S4 helices. (**b**) A hydrophobic cavity is formed by several aromatic and hydrophobic residues in pore helix 1, helices S5 and S6, and helix S6 of a neighbouring subunit (S6*). (**c**) A similar hydrophobic cavity is found in PKD2L1. (**d**) Sequence alignment of pore regions of PKD2L1 and TRPML1. Cavity residues of TRPML1 are labeled in green correspondingly.

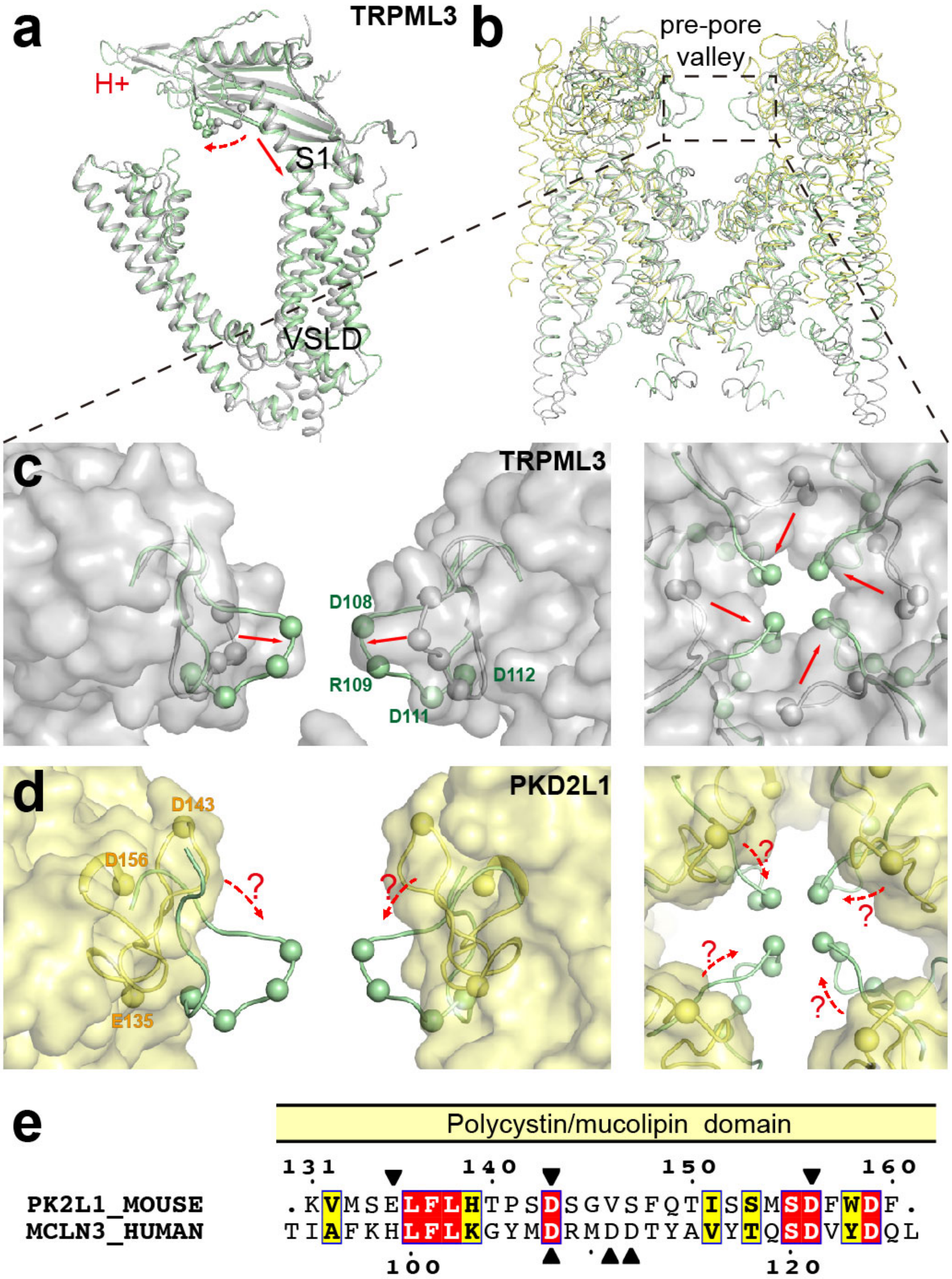

Supplementary Figure 8 Structural analysis of TRPML3's low-pH-induced conformational changes (closed-state apo-TRPML3 at pH 7.4 shown as gray, PDB code: 6AYE; inactivated-state apo-TRPML3 at pH 4.8 shown as pale green, PDB code: 6AYG)[12]. The red arrows represent overall shifts from the closed to inactivated state in response to low pH. (**a**) Structural comparisons of one protomer between

TRPML3's two states indicate its potential pH-regulated mechanisms. A big conformational change of the luminal pore loops transmits the pH signals to VSLD domains through the S1 helix. (**b**) The overall topology of PKD2L1 (pale yellow) and TRPML3 (gray and pale green) are parallel when superimposed. The pre-pore valley (defined in Main Text, Fig. 2) enclosed by luminal pore loops shows some vital changes in response to low pH. For visual clarity, only two diagonal protomers are shown. (**c**) The zoomed depiction of changeds in luminal pore loops in TRPML3, shown from the side view and top view respectively. Polar residues that may account for this pH sensitivity are highlighted as spheres. (**d**) Hypothetically, the off-response mechanism of PKD2L1 can be explained as likewise conformational changes of the luminal pore loops in the pre-pore valley area. These putative movements are shown in dotted arrows with question marks. There are also several polar residues on PKD2L1, highlighted as spheres. (**e**) Sequence alignments of luminal pore loops of PKD2L1 and TRPML3. Polar residues are marked with arrows correspondingly.

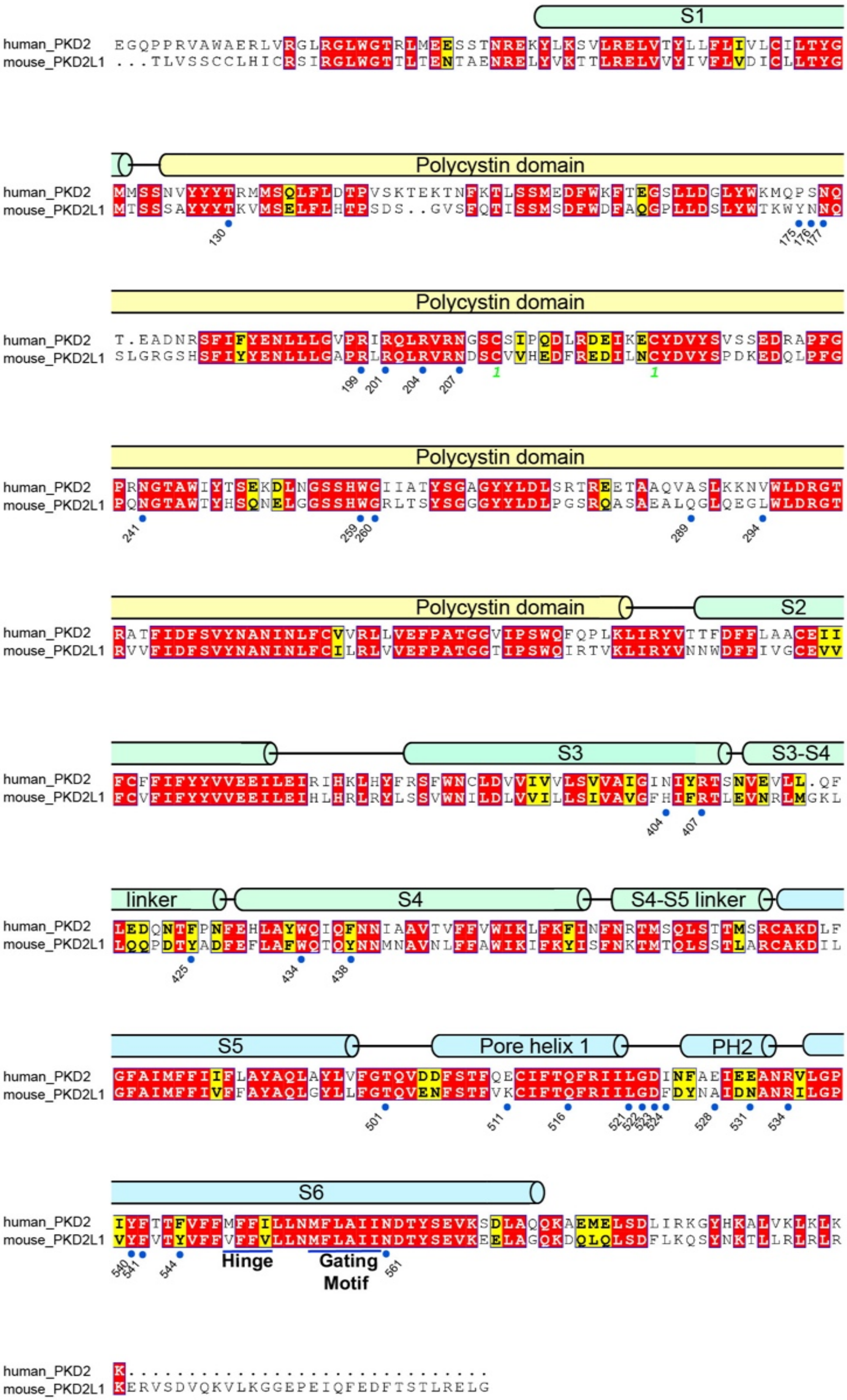
S1
human_PKD2 EGQPPRVAWAERLVRGLRGLWGTRLMEESSTNREKYLKSVLRELVTYLLFLIVLCILTYG
mouse_PKD2L1 ...TLVSSCCLHICRSIRGLWGTTLTENTAENRELYVKTTLRELVVYIVFLVDICLLTYG
Polycystin domain
human_PKD2 MMSSNVYYYTRMMSQLFLDTPVSKTEKTNFKTLSSMEDFWKFTEGSLLDGLYWKMQPSNQ
mouse_PKD2L1 MTSSSAYYYTKVMSELFLHTPSDS..GVSFQTISSMSDFWDFAQGPLLDSLYWTKWYNNQ
130
175
176
177
Polycystin domain
human_PKD2 T.EADNRSFIFYENLLLGVPRIRQLRVRNGSCSIPQDLRDEIKECYDVYSVSSEDRAPFG
mouse_PKD2L1 SLGRGSHSFIYYENLLLGAPRLRQLRVRNDSCVVHEDFREDILNCYDVYSPDKEDQLPFG
199
201
204
207
1
1
Polycystin domain
human_PKD2 PRNGTAWIYTSEKDLNGSSHWGIIATYSGAGYYLDLSRTREETAAQVASLKKNVWLDRGT
mouse_PKD2L1 PQNGTAWTYHSQNELGGSSHWGRLTSYSGGGYYLDLPGSRQASAEALQGLQEGLWLDRGT
241
259
260
289
294
Polycystin domain
S2
human_PKD2 RATFIDFSVYNANINLFCVVRLLVEFPATGGVIPSWQFQPLKLIRYVTTFDFFLAACEII
mouse_PKD2L1 RVVFIDFSVYNANINLFCILRLVVEFPATGGTIPSWQIRTVKLIRYVNNWDFFIVGCEVV
S3
S3-S4
human_PKD2 FCFFIFYYVVEEILEIRIHKLHYFRSFWNCLDVVIVVLSVVAIGINIYRTSNVEVLL.QF
mouse_PKD2L1 FCVFIFYYVVEEILEIHLHRLRYLSSVWNILDLVVILLSIVAVGFHIFRTLEVNRLMGKL
404
407
linker
S4
S4-S5 linker
human_PKD2 LEDQNTFPNFEHLAYWQIQFNNIAAVTVFFVWIKLFKFINFNRTMSQLSTTMSRCAKDLF
mouse_PKD2L1 LQQPDTYADFEFLAFWQTQYNNMNAVNLFFAWIKIFKYISFNKTMTQLSSTLARCAKDIL
425
434
438
S5
Pore helix 1
PH2
human_PKD2 GFAIMFFIIFLAYAQLAYLVFGTQVDDFSTFQECIFTQFRIILGDINFAEIEEANRVLGP
mouse_PKD2L1 GFAIMFFIVFFAYAQLGYLLFGTQVENFSTFVKCIFTQFRIILGDFDYNAIDNANRILGP
501
511
516
521
522
523
524
528
531
534
S6
human_PKD2 IYFTTFVFFMFFILLNMFLAIINDTYSEVKSDLAQQKAEMELSDLIRKGYHKALVKLKLK
mouse_PKD2L1 VYFVTYVFFVFFVLLNMFLAIINDTYSEVKEELAGQKDQLQLSDFLKQSYNKTLLRLRLR
540
541
544
561
Hinge
Gating Motif
human_PKD2 K.............................
mouse_PKD2L1 KERVSDVQKVLKGGEPEIQFEDFTSTLRELG

Supplementary Figure 9 Sequence alignment of mouse PKD2L1 and human PKD2. Human PKD2 aligned against PKD2L1 (residues 64–629) used in our research. Secondary structures are indicated as cylindric tubes over the sequence with the critical residues discussed in our paper highlighted. All important residues have been labeled in the figure as blue dots.

Supplementary Table 1. Data collection and model statistics

	PKD2L1
**Data collection**	
**EM equipment**	FEI Titan Krios
**Voltage (kV)**	300
**Detector**	Falcon 2
**Pixel size (Å)**	0.88
**Electron dose (e^-/Å^2)**	60
**Defocus range (μm)**	1.5~2.9
**Reconstruction**	
**Software**	RELION 1.4 & 2.0
**Number of particles used**	22,296
**Symmetry**	C4
**Final resolution (Å)**	3.38
**Map sharpening B-factor (Å^2)**	−170.923
**Accuracy of rotation (°)**	1.48
**Accuracy of translation (pixels)**	0.803
**Model building**	
**Software**	Coot
**Refinement**	
**Software**	Phenix
**Average Fourier shell correlation**	0.788
**Model composition**	
**Protein residues**	6864
**Side chains**	6748
**Sugar**	12
**Validation**	
**RMS deviations**	
**Bond lengths (Å)**	0.01
**Bond Angles (°)**	0.88
**Ramachandran plot statistics (%)**	
**Preferred**	95.51
**Allowed**	4.49
**Outlier**	0.00

Supplementary Table 2. Comparison of the pore size for selectivity filters and lower gates from different channels

Species	Protein	State	PDB ID	SF pore size in diagonal (Å)	Lower gate pore size in diagonal (Å)	Resolution (Å)
Mus musculus	PKD2L1	open	–	7.3	8.3	3.38
Homo sapiens	PKD2[1]	closed	5T4D	5.0	4.9	3.00
Rattus norvegicus	TRPV1[2]	open	5IRX	8.0	9.3	2.95
Rattus norvegicus	TRPV1[2]	closed	5IRZ	4.4	5.2	3.28
Rattus norvegicus	TRPV1[2]	semi-open	3J5R	4.2	7.3	4.20
Magnetococcus marinus	Na_vMs[3]	open	5HVX	7.6	7.8	2.45
Arcobacter butzleri	Na_vAb[4]	closed	3RVY	7.8	5.1	2.70
Arcobacter butzleri	Ca_vAb[5]	closed	5KLB	8.1	5.5	2.70
Oryctolagus cuniculus	$Ca_v1.1$[6]	closed	5GJV	5.2	4.7	3.60
Sus scrofa	RyR2[7]	open	5GOA	10.9	8.4	4.20
Sus scrofa	RyR2[7]	closed	5GO9	10.9	5.6	4.40

Supplementary Table 3. Primer sequences used in this study

Plasimid	Direction	Primer(5'-3')
PKD2L1_64_629_N3Flag_C2Strep	Forward	AAATATGCGGCCGCACCCTGGTGTCCAGCTGCT
	Reverse	CCGCTCGAGGTGCCCCAGTTCCCTCAAGG
PKD2L1_64_629_CYFP	Forward	CGGGGTACCGCCACCACCCTGGTGTCCAGCTGCT
	Reverse	AAATATGCGGCCGCGTGCCCCAGTTCCCTCAAGG
PKD2L1_1-760_CYFP	Forward	CGGGGTACCGCCACCATGAATAGTATGGAAAGC
	Reverse	AAATATGCGGCCGCGGACGGATTATACAGGTTCTCCCAAAC
PKD2L1_1-760_G522L_CYFP	Forward	GATAATCCTTCTTGATTTTGACTACAAT
	Reverse	ATTGTAGTCAAAATCAAGAAGGATTAT
PKD2L1_1-760_I560F_CYFP	Forward	GTTCCTGGCCATCTTCAACGACACATACTCCG
	Reverse	GTATGTGTCGTTGAAGATGGCCAGGAAC

Supplementary References

1. Shen, P.S. et al. The Structure of the Polycystic Kidney Disease Channel PKD2 in Lipid Nanodiscs. *Cell* **167**, 763-773 e11 (2016).
2. Cao, E., Liao, M., Cheng, Y. & Julius, D. TRPV1 structures in distinct conformations reveal activation mechanisms. *Nature* **504**, 113-8 (2013).
3. Sula, A. et al. The complete structure of an activated open sodium channel. *Nat Commun* **8**, 14205 (2017).
4. Payandeh, J., Scheuer, T., Zheng, N. & Catterall, W.A. The crystal structure of a voltage-gated sodium channel. *Nature* **475**, 353-8 (2011).
5. Tang, L. et al. Structural basis for Ca2+ selectivity of a voltage-gated calcium channel. *Nature* **505**, 56-61 (2014).
6. Wu, J. et al. Structure of the voltage-gated calcium channel Ca(v)1.1 at 3.6 A resolution. *Nature* **537**, 191-196 (2016).
7. Peng, W. et al. Structural basis for the gating mechanism of the type 2 ryanodine receptor RyR2. *Science* **354**(2016).
8. Wilkes, M. et al. Molecular insights into lipid-assisted Ca(2+) regulation of the TRP channel Polycystin-2. *Nat Struct Mol Biol* **24**, 123-130 (2017).
9. Grieben, M. et al. Structure of the polycystic kidney disease TRP channel Polycystin-2 (PC2). *Nat Struct Mol Biol* **24**, 114-122 (2017).
10. Liao, M., Cao, E., Julius, D. & Cheng, Y. Structure of the TRPV1 ion channel determined by electron cryo-microscopy. *Nature* **504**, 107-12 (2013).
11. Schmiege, P., Fine, M., Blobel, G. & Li, X. Human TRPML1 channel structures in open and closed conformations. *Nature* **550**, 366-370 (2017).
12. Zhou, X. et al. Cryo-EM structures of the human endolysosomal TRPML3 channel in three distinct states. *Nat Struct Mol Biol* **24**, 1146-1154 (2017).
13. Biasini, M. et al. SWISS-MODEL: modelling protein tertiary and quaternary structure using evolutionary information. *Nucleic Acids Res* **42**, W252-8 (2014).
14. Pettersen, E.F. et al. UCSF Chimera--a visualization system for exploratory research and analysis. *J Comput Chem* **25**, 1605-12 (2004).
