# Supplementary material for: Cryo-EM structure of the polycystic kidney disease-like channel PKD2L1
Source: Nat Commun. 2018 Mar 22;9:1192. doi: 10.1038/s41467-018-03606-0 (PMC5864754; doi:10.1038/s41467-018-03606-0)
Supplement: Supplementary file 3 — Description of Additional Supplementary Files(PDF 6 kb) [file 41467_2018_3606_MOESM3_ESM.pdf]

Supplementary Movie Legends

Supplementary Movie 1 Overall structural differences between PKD2L1 and its homologous model. We generated a homologous model of closed-state PKD2L1 based on the structure of PKD2 following sequence alignment using the SWISS-MODEL website (https://www.swissmodel.expasy.org). The modelled structure was superimposed to the cryo-EM structure of PKD2L1 with respect to the whole structures. The homologous structural model of PKD2L1 and the cryo-EM structure of PKD2L1 were used respectively as the initial and end frame for morph generation, representing closed- and open-state PKD2L1. The intermediate morphs were generated using Chimera. The movies were prepared in PyMOL. The structures are domain colored following the same scheme as in Fig. 1. The same was applied to Supplementary Movies 2 and 3. Specific views are shown in each movie. In the side views, the luminal side is placed at the bottom.

Supplementary Movie 2 Conformational differences of the selectivity filter and lower gate between PKD2L1 and its homologous model. Changes are shown from an extracellular view. The conserved $L^{521}GD^{523}$ in PKD2L1 (resp. $L^{641}GD^{643}$ in PKD2) on the selectivity filter are shown as sticks. The comparison of the upper gate between the ion-conducting state (PKD2L1) and the non-conducting state (PKD2L1 closed-state homologous model) suggests a dilation of the upper gate with each pore helix moving away from the central axis of the pore. The axial rotations of S6 segments between the two structures result in the placement of the conserved constriction residues inside or outside the lower gate.

Supplementary Movie 3 Conformational differences of each domain between PKD2L1 and its homologous model. The rotations of the VSDs and pore regions are evident in the side view. For visual clarity, only two diagonal protomers are shown.
